# Diamond Nanoparticles-Porphyrin mTHPP Conjugate as Photosensitizing Platform: Cytotoxicity and Antibacterial Activity

**DOI:** 10.3390/nano11061393

**Published:** 2021-05-25

**Authors:** Carolina Ramos Hurtado, Gabriela Ramos Hurtado, Gabrielle Lupeti de Cena, Rafaela Campos Queiroz, Alexandre Vieira Silva, Milton Faria Diniz, Verônica Ribeiro dos Santos, Vladimir Trava-Airoldi, Maurício da Silva Baptista, Ncediwe Tsolekile, Oluwatobi Samuel Oluwafemi, Katia Conceição, Dayane Batista Tada

**Affiliations:** 1 Federal Institute of São Paulo (IFSP), São José dos Campos 12223-201, São Paulo, Brazil; carolina.hurtado@ifsp.edu.br (C.R.H.); rafaelacamposqueiroz003@gmail.com (R.C.Q.); 2 Nanomaterials and Nanotoxicology Laboratory, Institute of Science and Technology, Federal University of São Paulo (UNIFESP), São José dos Campos 12231-280, São Paulo, Brazil; 3 Peptide Biochemistry Laboratory, Institute of Science and Technology, Federal University of São Paulo (UNIFESP), São José dos Campos 12231-280, São Paulo, Brazil; gabrielle.lupeti@unifesp.br (G.L.d.C.); katia.conceicao@unifesp.br (K.C.); 4 Institute of Science and Technology, São Paulo State University (UNESP), São José dos Campos 12247-004, São Paulo, Brazil; gabriela.hurtado@ict.unesp.br; 5 Institute of Advanced Sea Studies (IEAMAr), São Paulo State University (UNESP), São José dos Campos 12247-004, São Paulo, Brazil; 6 Institute of Medicinal Chemistry, Helmholtz Zentrum München, 30167 Hanover, Germany; silva.alexandre.v@gmail.com; 7 Fundamental Sciences Division, Technological Institute of Aeronautics (ITA), São José dos Campos 12228-900, São Paulo, Brazil; mfariadiniz@gmail.com; 8 Bioceramics Laboratory, Institute of Science and Technology, Federal University of São Paulo (UNIFESP), São José dos Campos 12231-280, São Paulo, Brazil; veronica.ribeiro@unifesp.br; 9 Sensors and Materials Associated Laboratory, National Institute for Space Research (INPE), São José dos Campos 12227-010, São Paulo, Brazil; vladimir.airoldi@inpe.br; 10 Department of Biochemistry, Institute of Chemistry, University of São Paulo (USP), São Paulo 05508-000, São Paulo, Brazil; baptista@iq.usp.br; 11 Department of Chemical Sciences, University of Johannesburg, P.O. Box 17011, Doornfontein, Johannesburg 2028, South Africa; nncediwe@gmail.com (N.T.); ooluwatobi@uj.ac.za (O.S.O.); 12 Centre for Nanomaterials Science Research, University of Johannesburg, Johannesburg 2028, South Africa

**Keywords:** photosensitizers (PS), nanoconjugate, photodynamic antimicrobial therapy (aPDT), drug-resistance

## Abstract

Conjugation of photosensitizers (PS) with nanoparticles has been largely used as a strategy to stabilize PS in the biological medium resulting in photosensitizing nanoparticles of enhanced photoactivity. Herein, (Meso-5, 10, 15, 20-tetrakis (3-hydroxyphenyl) phorphyryn (mTHPP) was conjugated with diamond nanoparticles (ND) by covalent bond. Nanoconjugate ND-mTHPP showed suitable stability in aqueous suspension with 58 nm of hydrodynamic diameter and Zeta potential of −23 mV. The antibacterial activity of ND-mTHPP was evaluated against *Escherichia coli* for different incubation times (0–24 h). The optimal activity was observed after 2 h of incubation and irradiation (660 nm; 51 J/cm^2^) performed right after the addition of ND-mTHPP (100 μg/mL) to the bacterial suspension. The inhibitory activity was 56% whereas ampicillin at the same conditions provided only 14% of bacterial growth inhibition. SEM images showed agglomerate of ND-mTHPP adsorbed on the bacterial cell wall, suggesting that the antimicrobial activity of ND-mTHPP was afforded by inducing membrane damage. Cytotoxicity against murine embryonic fibroblast cells (MEF) was also evaluated and ND-mTHPP was shown to be noncytotoxic since viability of cells cultured for 24 h in the presence of the nanoconjugate (100 μg/mL) was 78%. Considering the enhanced antibacterial activity and the absence of cytotoxic effect, it is possible to consider the ND-mTHPP nanoconjugate as promising platform for application in antimicrobial photodynamic therapy (aPDT).

## 1. Introduction

Diamond nanoparticles (ND) are carbon-based materials that have attracted significant attention due to their excellent mechanical properties, chemical inertia of the diamond core, high biocompatibility and fluorescence when nitrogen vacancy centers are present in their crystalline lattice [1,2,3,4,5,6]. The application of ND in the most diverse field of materials engineering has been further expanded by ND surface functionalization with reactive chemical groups affording the modulation of hydrophobicity and (bio) molecular interaction. 

Depending on the synthetic route, ND may have different surface chemical groups. The most commonly techniques to produce ND are high pressures and high temperatures (HPHT) [7], plasma-assisted chemical vapor deposition (PACVD), hot filament chemical vapor deposition (HFCVD), explosive detonation [7], laser ablation [3,8,9], and ultrasonic cavitation [10,11]. Recently ND with hydrogenated surface were obtained from leftover of HFCVD system by ultrasonic cavitation [12] and high-energy mill grinding [13]. The ND obtained by explosive detonation have high surface density of oxidized chemical groups that are highly reactive and enable the coupling of different types of molecules [14,15,16,17]. The functionalization of ND by adsorption or covalent bond with drugs, proteins, peptides, nucleic acids, make them an emerging class of drug delivery platforms. Moreover, ND containing oxidized [18] and partially oxidized [19] surface have shown high affinity with cellular components affording antibacterial activity against several strains such as *Staphylococcus aureus*, *Streptococcus mutans*, *Bacillus subtilis*, and *Escherichia coli* [19,20,21]. It is expected that the antimicrobial activity of these ND is afforded by the hydrogen bond between the surface group of the ND and the lipids from the bacterial cell wall. Cao et al. (2018) [20] showed evidence of hydrogen bonds between the 2-hydroxyethylmethacrylate (HEMA) from surface-functionalized ND and the lipid head groups from the bacterial cell wall. Consequently, lipid membrane fluidity and selectivity were disrupted, compromising bacterial growth. 

Most of the ongoing research on the development of antimicrobial agents as alternatives to the commonly used antibiotics have targeted the microorganisms from ESKAPE group (*Enterococcus* spp., *S. aureus, Klebsiella pneumoniae*, *Acinetobacter baumannii*, *Pseudomonas aeruginosa* and *Enterobacter* spp., such as *E. coli*). ESKAPE group has demanded significant attention for having developed drug-resistance and became a worldwide health problem. *E. coli*, for example, is mostly harmless, but under abnormal growth it can cause serious diseases in humans, such as gastroenteritis and infections in the urinary tract, bloodstream and central nervous system. In an attempt to minimize the problem of drug resistance, some alternatives have been considered, such as the development of new antibiotics [22] or the development of alternative therapies such as the therapy based on the use of bioactive peptides [23,24,25]; phytochemicals or nanoparticles [26,27,28]. Antimicrobial photodynamic therapy (aPDT) is another example of alternative therapy that has shown encouraging results in experimental and clinical applications. 

Porphyrins and other photosensitizers (PS) that were known for their high efficiency in PDT of cancer have also been widely investigated for application in aPDT [29,30,31,32]. In comparison with conventional antimicrobial approaches, aPDT offers the advantage of lower incidence of microbial resistance [30,31,33]. The efficiency of a PS as photoactive molecule for aPDT depends on their interaction with bacterial cell wall, cell uptake as well as on their quantum yield of ROS generation upon light irradiation, since these are the toxic species responsible for leading microorganisms to death. Nevertheless, although several PS have shown high antimicrobial and/or antitumoral activity in vitro, the deactivation of PS in the biological medium and their aggregation has been identified as the most critical factors that compromise their photoactivity in vivo. In order to overcome these drawbacks faced by the use of free PS, a large effort has been dedicated to the development of photosensitizing nanoparticles by coupling PS molecules to nanoparticles surface [34,35,36,37].

Considering the antimicrobial activity of the ND and their reactive surface chemistry, this work addresses the coupling of ND with porphyrin aiming at the development of highly efficient antimicrobial agent. Despite the innumerous studies on the use of ND in biomedical applications, the combined use of ND and PS has been almost unstudied, but the little information reported until now has highlighted that it is a promising approach to enhance therapeutic efficiency of PDT of cancer and of aPDT. Recently, Matshitse et al. (2020) [38] reported on the coupling of 2,9(10),16(17),23(24)-tetrakis-(4-pyridyloxy)phthalocyanato-silicon(IV)hydroxide (Si(OH)_2_TPPc) (ZnTPPcQ) with detonation ND by covalent bond and evaluated antitumoral effect against breast cancer cells (MCF7). The conjugate reduced cell viability to 29% after irradiation and at the concentration of 50 μg/mL. Herein, the porphyrin (Meso-5, 10, 15, 20-tetrakis (3-hydroxyphenyl), mTHPP was conjugated with detonation ND by covalent bond (esterification). The developed nanoconjugate ND-mTHPP proved to be stable in aqueous suspension with hydrodynamic diameter of 58 nm and Zeta potential of −23 mV. The inhibitory activity against *E. coli* was of 56% after 2 h of incubation and under irradiation (660 nm, 51 J/cm^2^). Remarkable, the inhibition of *E. coli* growth performed by ampicillin was only 26% in the same conditions.

The advances of nanotechnology had raised the concerns about the impact of nanomaterials in health and environment. It has been increasingly clear that toxicity assays have to be part of the development of a nanomaterial. For this reason, the cytotoxicity of ND-mTHPP was investigated in this work by in vitro assay with MEF cells in the presence and absence of light irradiation (660 nm; 51 J/cm^2^). ND-mTHPP was shown to be noncytotoxic against MEF cells in the absence of irradiation. To the best of our knowledge, this is the first work reporting on the covalent binding of porphyrin to ND and on the evaluation of the antimicrobial and cytotoxic activity of the nanoconjugate. The low cytotoxic and the expressive antibacterial activity renders the ND-mTHPP conjugate a promising application in aPDT.

## 2. Materials and Methods

### 2.1. Diamond Nanoparticles and Organics

Detonation diamond nanoparticles were obtained from iTC—International Technology Center (Durham, NT, USA), Standard Nanodiamond. Dulbecco’s Modified Eagle Medium (DMEM) (Gibco) was prepared in deionized water and buffered with sodium bicarbonate (Synth, Brazil) and supplemented with fetal bovine serum (FBS) (Vitrocell Embriolife). Streptomycin, ampicillin, 3-(4,5dimethylthiazol-2-yl)-2,5-diphenyltetrazolium bromide (MTT), and dimethylsulphoxide (DMSO) were purchased from Sigma-Aldrich (St. Louis, MO, USA). Mueller Hinton Broth Medium (MHB) (Kasvi) was prepared in deionized water. Sulfuric, nitric and hydrochloric acids were purchased from Synth (Brazil). 1-ethyl-3-[3-dimethylaminopropyl] carbodiimide hydrochloride (EDC), and N-hydroxysuccinimide (NHS) were purchased from Sigma-Aldrich. All reagents and solvents were used as received from commercial suppliers.

### 2.2. ND Purification

The commercially obtained ND powder was cleansed by two acid treatments which also provided oxidation of chemical surface groups. A mass of 0.2 g of ND was stirred for 2 h with 10 mL of HNO_3_: HCl (1:3). After this period, the decanted powder was washed with ultrapure water until the suspension was neutralized. Then, the ND was dried in an oven at 100 °C for 24 h. Following, the powder was stirred with 10 mL of H_2_SO_4_: H_2_O_2_ (4:1) for 2 h. After this period, the decanted powder was washed with ultrapure water until the suspension was neutralized. The obtained powder was dried in an oven at 100 °C for 24 h. 

### 2.3. Synthesis of meso-5, 10, 15, 20-Tetrakis (3-Hydroxyphenyl) Porphyrin—(mTHPP)

The mTHPP was synthesized by following the protocol reported by Tsolekile et al. (2018) [37]. Typically, an equimolar solution of 3-hydroxybenzaldehyde in propionic acid (1:1, *v/v*) was stirred for 15 min at 45 °C. Then 1.42 mL of pyrrole was quickly added. The resulting mixture was stirred under reflux for an additional 1.5 h. After that, the crude was allowed to reach room temperature and the propionic acid was evaporated and, at room temperature, the residue was neutralized with NaHCO_3_ and the precipitated crude porphyrin was washed with chloroform, dried over Na_2_SO_4_ and concentrated under reduced pressure. The obtained mTHPP was further purified by column chromatography (SiO_2_) using ethyl acetate:hexane (2:1, *v/v*) as eluent.

### 2.4. Synthesis of ND-mTHPP Nanoconjugate System

**Figure 1:**
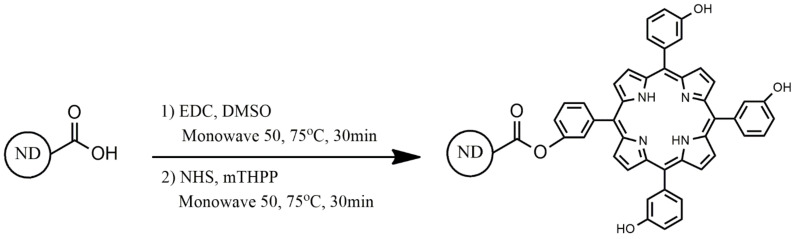
Schematic representation (out of scale) of the conjugation of mTHPP with ND-COOH through esterification resulting in ND-mTHPP nanoconjugate system.

ND-COOH (5 mg) was suspended in DMSO (3 mL) in a G10 glass reaction flask containing a magnetic PTFE bar. Then, EDC (5 mg) was added to the solution. The flask was sealed with PTFE septum and a specific cap for G10. The system was placed in a pressurized reactor of conventional heating synthesis, Monowave 50 (Anton Paar), under magnetic stirring, at 75 °C for 30 min followed by addition of mTHPP solubilized in DMSO (0.3 mg/800 μL) and NHS (5 mg) and the reaction mixture was further stirred at 75 °C for 30 min in reactor. After this period, the flask was centrifuged at 4000× *g* for 5 min. The nanoconjugate powder systems were washed several times with ethanol, followed by centrifugation steps and the recovered powder was dried at 75 °C for 48 h and called herein ND-mTHPP nanoconjugate (Figure 1).

### 2.5. Size and Zeta Potential—DLS (Dynamic Light Scattering)

The zeta potential, hydrodynamic diameter and particle size distribution of the ND samples were analyzed by dynamic light scattering using a Beckman-Coulter Delsa Nano C zeta-sizer analyzer (Beckman Coulter Inc., Brea, CA, USA). Particle size distribution was measured by using ND-COOH and ND-mTHPP aqueous suspensions (100 μg/mL). Particle size distribution of ND-mTHPP suspension in culture medium was also measured in order to evaluate the influence of culture media (MHB and DMEM) on ND colloidal stability. In this assay, ND-mTHPP was firstly suspended in water and then, the suspension was diluted in each culture medium at the same conditions used in the cell viability and antibacterial activity assays.

### 2.6. FT-IR (Fourier Transformed Infrared Spectroscopy)

The infrared spectra were acquired by Fourier transform infrared spectroscopy using a universal attenuated total reflectance sensor (FT-IR-UATR) (Perkim Elmer Spectrum, model Frontier, Waltham, MA, USA). The FT-IR spectrum was an average of 32 scans at a speed of 2 s per scan in a range of 400–4000 cm^−1^. The resolution of the spectrometer was of 4 cm^−1^. 

### 2.7. Cell Viability Assay

Murine Embryonic Fibroblast cells (MEF) were cultured in Dulbecco’s Modified Eagle’s Medium (DMEM) supplemented with 20% (*v/v*) FBS, sodium bicarbonate (2 g/L), streptomycin (0.1 g/L), and ampicillin (0.025 g/L). Cells were incubated at 37 °C in a humidified atmosphere with 5% CO_2_. MEF cells were seeded into 96-well plates (1 × 10^4^ cells per-well) and incubated for 24 h. Then, the culture medium was removed and replaced by fresh culture medium containing the different samples. Cells were treated with ND-mTHPP nanoconjugated system (100 µg/mL) and ND-COOH (100 µg/mL) prepared in ultrapure water. After treatment, the plates were irradiated (51 J/cm^2^) by using an LED-coupled irradiation chamber (IrradLed, Biopdi), with maximum emission at 660 nm. The irradiated cells and the nonirradiated cells were further incubated for 24 h. After 24 h, the cells were washed with 100 µL fresh PBS. The cell viability was evaluated by incubating the cells with 100 µL of a MTT (3-(4,5dimethylthiazol-2-yl)-2,5 diphenyl tetrazolium bromide) solution (0.5 mg/mL) for 3 h. Thereafter, MTT solution was replaced by DMSO to solubilize formazan crystals. The final absorbance of formazan was measured in a microplate reader (Synergy H1-Biotek) at 540 nm. The absorbance of cells incubated without samples and in the absence of irradiation were considered as 100% of viability. The cell viability data were analyzed by 1-way ANOVA and Tukey’s multiple range (*p* < 0.05) to determine statistical differences between different groups of samples.

### 2.8. Antibacterial Assay

The antimicrobial activity was evaluated by using a modified NCCLS broth microdilution method (Pfaller et al. 2002) [39]. Antimicrobial activity was monitored using a liquid growth inhibition assay against *E. coli* (ATCC 25922). The pre-inoculum of the strain was prepared in MHB (Mueller Hinton Broth Medium) for approximately 12 h at 37 °C. The inoculum was standardized as 10^8^ cells/mL by measuring absorbance at 600 nm and plated into 96-well plates. The 50 µL of ND suspensions (ND-mTHPP—100 µg/mL) or ND-COOH (100 µg/mL) prepared in ultrapure water were added in each well containing 200 µL of inoculum. Following, a group of plate was irradiated (51 J/cm^2^) by using an LED-coupled irradiation chamber (IrradLed, Biopdi), with maximum emission at 660 nm. The irradiated *E. coli* plate and the nonirradiated *E. coli* plate were incubated for a total of 24 h at 37 °C. The inhibition of bacterial growth was evaluated by measuring absorbance at 600 nm at different periods after the irradiation from 0 to 24 h (Synergy H1-Biotek). For comparative purpose, the absorbance at 600 nm of the nonirradiated plate was performed at the same time as the irradiated plate. Ampicillin (Sigma-Aldrich) was used as a positive control of inhibition growth. The *E. coli* grown in MHB in the absence of any samples was used as a negative control. 

In order to evaluate the interaction of ND-mTHPP with the bacteria by scanning electron microscopy (SEM), aliquots (10 µL) of bacterial suspension at the end of the antibacterial assay were dropped on glass slides. Following, the glass slides were placed inside petri dishes and 500 µL of methanol were added on each glass slide and left to rest for 1 h. Then, the liquid was removed and subsequent addition/remotion cycles of ethanol/water solution at increasing concentration of ethanol were performed. Volumes of 500 mL of solutions containing from 10% to 90% of ethanol were added and left to rest on each glass slide for 20 min. Then, 500 µL of ethanol (100%) was added on each glass slide and left to rest for 1 h. After removing ethanol, the glass slides dried at room temperature inside the petri dishes. The slides were sputter coated with gold for 90 s at 20 mA (Sputtering Quorum Q150R ES) before being imaged by SEM (FEI Inspect S50, Hillsboro, OR, USA) available at NAPCEM-UNIFESP.

## 3. Results

### 3.1. Conjugation of mTHPP with ND

**Figure 2:**
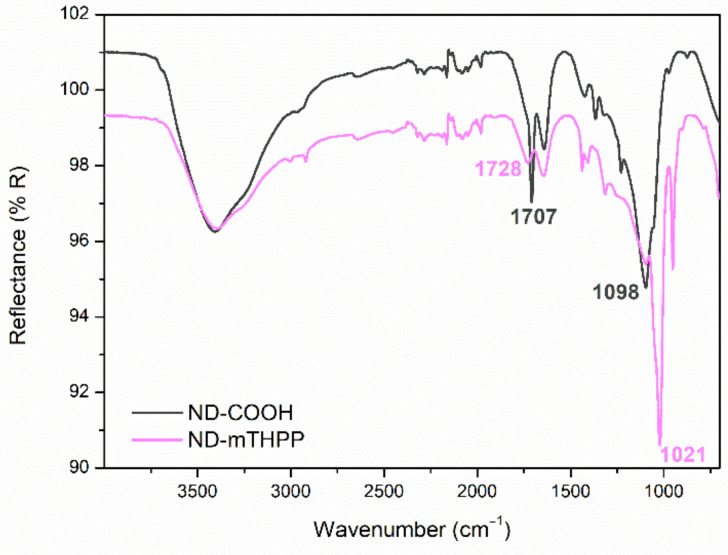
FT-IR spectroscopy ND-COOH and conjugated system (ND-mTHPP).

The powder of ND resultant from the purification step was named ND-COOH, since the process was expected to produce ND with oxidized surface. Then, ND-COOH was used as starting material for the synthesis of the ND-mTHPP nanoconjugate via esterification as represented in Figure 2. The success of the conjugation could be evidenced by comparative analysis between the FT-IR spectrum of the starting material (ND-COOH) and the FT-IR spectrum of the final product (ND-mTHPP) as depicted in Figure 2. An intense band at 1707 cm^−1^ corresponds to the stretching vibrational mode of the C=O bond of the carboxyl of the carboxylic group from the ND-COOH surface. This band was shifted to a higher wave number, 1728 cm^−1^, in the spectrum of nanoconjugate ND-mTHPP, which could be assigned to the stretching vibrational mode of the C=O bond of the ester bond. Another piece of evidence of the ester bond formation was the shift of the band at 1098 cm^−1^ (ND-COOH) to smaller wave number (1021 cm^−1^) after the conjugation, which represents the change from the vibrational modes of the C–O bond of carboxylic acids to the C–O of the ester chemical group. Furthermore, it was also possible to observe the bands at 2921 and 3006 cm^−1^ which correspond to the mTHPP pyrrolic N-H.

Similar results were obtained by Matshitse et al. (2019) [40] who reported on the formation of conjugate system between detonation nanodiamond and ZnTPPcQ by a covalent bond. The conjugation by covalent bond was confirmed by FT-IR, wherein it was observed the shift of the band close to 1700 cm^−1^ (attributed to the stretching vibrational mode of the C=O bond of the carboxylic group present in the ND surface) to the region around of 1600 cm^−1^, that could be associated with the presence of RCOOR, due to the formation of the covalent bond of the phthalocyanine hydroxyl group and the carboxyl groupd from ND. Recently, Tsolekile et al. (2020) [41] reported on the covalent bond between ternary quantum dots (CuInS/ZnS) capped with glutathione and mTHPP porphyrin, via an esterification reaction as proven by the band at 1015 cm^−1^, in the FT- IR spectrum of the conjugate, which has been assigned to the vibrational mode of the C–O ester bond. 

### 3.2. ND Size, Zeta Potential and Size Distribution

**Figure 3:**
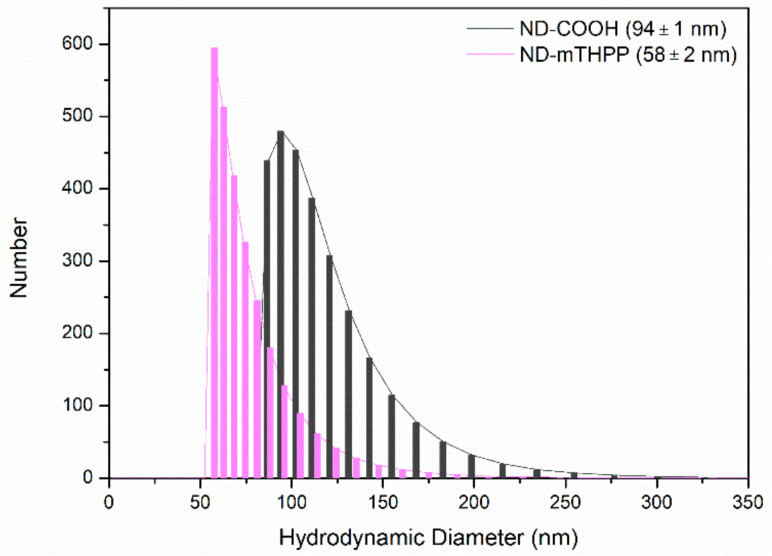
DLS size distribution of ND-COOH and ND-mTHPP in aqueous suspension (100 μg/mL).

The median hydrodynamic diameter (D) and the size distribution of the ND were measured before and after the conjugation with mTHPP. The median value of D after the conjugation was lower (D = 58 ± 2 nm) than the median D measured for ND-COOH (D = 94 ± 1 nm), which presented twice the value of D of ND-mTHPP. It was also observed that ND-mTHPP dispersed in aqueous medium (Figure 3) generates a suspension with a narrower size distribution (FWHM = 23) in comparison with the aqueous suspension prepared with ND-COOH (FWHM = 45). These results indicated that functionalization with mTHPP disrupted ND-COOH agglomerates and enhanced colloidal stability of the ND in suspension. The high value of Zeta potential (−23 mV) of ND-mTHPP indicated that the higher colloidal stability was a result of the increased electrostatic repulsion between the particles. 

The particle size of nanoparticles is critical to their cell uptake and interaction with membranes. In general, the surface chemistry and composition of nanoparticles was proven to change in the biological medium. Proteins and other biomolecules are known to adsorb on the surface of nanoparticles, affording their biological identity [18,42,43]. The surface coating composed by the biomolecules may change colloidal stability and nanoparticles interaction with cells. Thus, the D of the ND-mTHPP nanoconjugate was evaluated in the present work in the different media used in the in vitro assay: the MHB medium used in the antimicrobial assay and the DMEM medium supplemented with 20% SFB, used in the cytotoxicity assay (Table 1). It was not possible to notice significant difference between values of D measured with ND-mTHPP suspended in H_2_O (D = 58 ± 1 nm) and suspended in MHB (D = 58 ± 2 nm). However, increased size was measured when ND-mTHPP was suspended in DMEM (D = 82 ± 3 nm). In a similar study, Hamelaar et al. (2017) [44] showed increased size of ND in DMEM supplemented with 10% FBS. The authors also noticed that aggregation process depended on the order of the addition. When the ND were dispersed directly in DMEM-10% FBS, ND formed aggregates with D values around 860 nm. Nevertheless, when the ND were first dispersed in FBS, followed by the addition of DMEM, the measured values of D were of 90 nm, a value very similar to the D value measured herein (D = 82 nm). According to the authors, this procedure provides a thin layer of proteins on the ND surface preventing aggregation when ND were added in DMEM, which is a medium rich in salts.

### 3.3. Antibacterial Assay

The antibacterial effect of ND-mTHPP was evaluated against *E. coli*, a pathogenic microorganism commonly found in the intestine of humans and other mammalian animals, which can be beneficial or harmful to health, depending on where it is installed in the body. Usually, *E. coli* causes health problems if it invades other tissues or body fluids, such as blood and urine.

**Figure 4:**
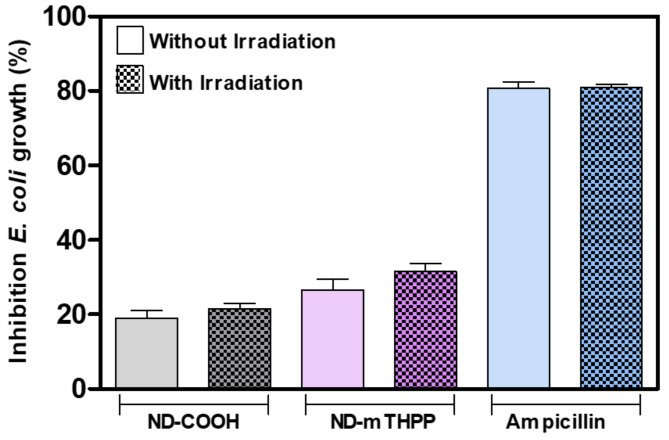
Percentage of inhibition of *Escherichia coli* growth calculated by considering OD_600_ of *E. coli* grown in the absence of sample and irradiation as 100% of growth. *E. coli* was incubated with ND-COOH (100 μg/mL), ND-mTHPP (100 μg/mL) and ampicilin (100 μg/mL), in Mueller Hinton Broth Medium (MHB) for 24 h, with and without irradiation (660 nm; 51 J/cm^2^). Experiments were repeated in triplicate and all the values were statistically different (*p* < 0.001) in comparison with control group of *E. coli* growth (without sample and in the absence of irradiation).

For comparative purposes, once in the synthesis of ND-mTHPP a previous ND surface functionalization with carboxyl groups is required, the ND-COOH was also evaluated regarding its antibacterial activity after 24 h incubation with *E. coli* (Figure 4). The % of *E. coli* growth in the presence of each type of ND and in the presence of antibiotic ampicillin indicated distinct inhibitory activities. The results revealed a moderated antibacterial activity of both ND-mTHPP and ND-COOH but when the irradiation was performed right after the incubation, the antibacterial activity of ND-mTHPP was enhanced (31 ± 5% of growth inhibition) in comparison with ND-mTHPP in the absence of irradiation (27 ± 6% of growth inhibition) and also in comparison with irradiated ND-COOH (22 ± 1% of growth inhibition). Nevertheless, both samples, ND-mTHPP and ND-COOH, showed relatively low antimicrobial activity when compared with ampicillin at the same concentration (100 μg/mL). 

Although most of the experimental protocols of antimicrobial assays include the OD_600_ measurement after 24 h of incubation of microorganism with the samples under study, a comprehensive study on the works wherein antimicrobial activity of ND was addressed pointed out that it was commonly evaluated by using shorter incubation time. Lyer et al. (2018) [45] for example, reported 50% of inhibition of the growth of Uropathogenic *E. coli* (UPEC) by incubation with 6 nm detonation ND at 200 μg/mL for 2 h. Chatterjee et al. (2016) [18] evaluated the antimicrobial activity of 5 nm detonation ND at 100 μg/mL, estimating the growth of *E. coli* (HB101) by OD_600_ measurements after 13 h. Wheling et al. (2018) [19] also observed an antibacterial effect (90% of growth inhibition) in the first 15 min of contact between 60 nm (100 μg/mL) detonation ND with *E. coli* K12 (DSMZ No. 1077). 

**Figure 5:**
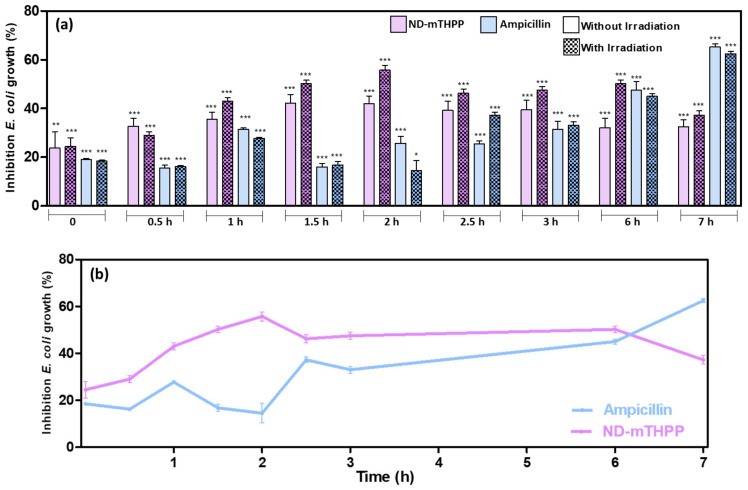
Inhibition of *Escherichia coli* growth. (**a**) Percentage of growth was calculated for *E. coli* incubated with ND-mTHPP (100 μg/mL) and *E. coli* incubated with Ampicillin (100 μg/mL), in Mueller Hinton Broth Medium (MHB), with and without irradiation at different time after the irradiation. (**b**) Percentage of growth was calculated for *E. coli* incubated with ND-mTHPP (100 μg/mL) and *E. coli* incubated with Ampicilin (100 μg/mL), in Mueller Hinton Broth Medium (MHB), with irradiation. Experiments were performed in triplicate and significant differences between *E. coli* control and test samples are marked with * (*p* < 0.05), ** (*p* < 0.01), and *** (*p* < 0.001).

Therefore, considering the different incubation time reported on the aforementioned works, the antibacterial activity of ND-mTHPP was further investigated herein as a function of incubation time. The bacterial growth was monitored for 7 h. Figure 5a shows the average and standard deviation values of % of bacterial growth calculated from the values of OD_600_ measured after each period of incubation of *E coli* with the ND-mTHPP and positive and negative controls. The values of % of growth inhibition in the presence and absence of irradiation are depicted in Table 2. It was possible to note the increasing growth inhibition by ND-mTHPP in the initial phase of bacterial growth (2 h). In this period, the % of growth inhibition increased from 24 ± 14% to 42 ± 6% after 2 h of incubation with ND-mTHPP. Remarkably, the value of % of growth inhibition promoted by ampicillin after 2 h was statistically different and lower (26 ± 6%) than the value found after 2 h of incubation with ND-mTHPP (42 ± 6%). 

The results suggested that the ND-mTHPP nanoconjugate had better antibacterial activity during 2 h of incubation than ampicillin, which is one of the antibiotics applied to treat infections caused by *E. coli*. It is noteworthy that this result was obtained with the ND-mTHPP conjugate even in the absence of irradiation, which is not commonly observed with the use of photosensitizing systems although it has already been described for porphyrins [46]. The comparison between the *E. coli* treatment with ND-mTHPP and with ampicillin can be better visualized in Figure 5b. It was clearly shown that under irradiation, the ND-mTHPP was proven to be even more efficient, promoting 56 ± 4% of growth inhibition of *E. coli* after 2 h of incubation whereas ampicillin promoted only 14 ± 9% of growth inhibition. It was observed that the growth of *E. coli* incubated with ND-mTHPP and irradiated was resumed after 2 h, thus showing a decrease in growth inhibition to 47 ± 3 and 50 ± 3% after 3 and 5 h, respectively. After 7 h of incubation, *E. coli* growth inhibition in the presence of ND-mTHPP was 37 ± 4%, while in the presence of ampicillin, *E. coli* showed 62 ± 2% growth inhibition.

Since the irradiation did not change bacterial growth in the negative control, the enhanced antimicrobial activity of ND-mTHPP observed after 2 h of incubation following the irradiation was not an isolated effect of the irradiation and therefore this result pointed to the suitable photoactivity of the ND-mTHPP nanoconjugate. 

**Figure 6:**
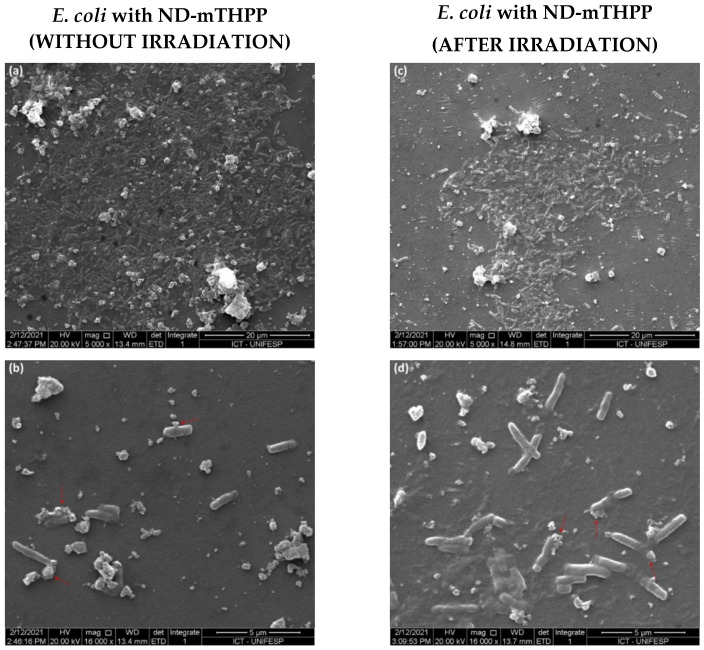
Images of SEM of *Escherichia coli* grown in the presence of ND-mTHPP in the absence of irradiation (**a**,**b**) and after irradiation (660 nm; 51 J/cm^2^) (**c**,**d**).

The SEM analysis of the *E. coli* after incubation with ND-mTHPP provided further insights regarding the mechanism through which ND-mTHPP performed antibacterial activity. The images (Figure 6) showed big agglomerates of ND-mTHPP on the cell wall of the bacteria. Since the results of DLS pointed to the high colloidal stability of the ND-mTHPP nanoconjugate, it is expected that these agglomerates were formed under the drying process during the sample preparation for SEM analysis or upon their interaction with the bacterial cell wall. In fact, the agglomeration of ND and other nanoparticles upon the adsorption on cell membranes or on bacterial cell wall was already reported by several researchers [18,47]. The presence of these agglomerates would promote loss of membrane integrity, selectivity and increased fluidity which lead to bacteria death. The damage in the cell wall could be clearly observed in the high-magnification images. The red arrows in the images highlighted the damaged portion of the bacteria which colocalized with the ND-mTHPP agglomerates. Therefore, these observations supported our claim that the antimicrobial activity was mainly a result of the interaction between ND-mTHPP with lipid membrane. Furthermore, although a colony of bacteria was still observed after irradiation, the size of the colony was smaller than the observed bacteria colony without irradiation. Under irradiation, ND-mTHPP was expected to generate ROS, in association with the effect of ND-mTHPP adsorption on the cell wall that led to enhanced toxicity of ND-mTHPP against *E. coli*.

### 3.4. Cell Viability Assay

The cytotoxicity of ND-mTHPP was investigated in this work by in vitro assay with MEF cells in the presence and absence of light irradiation (660 nm; 51 J/cm^2^). Since the ND-COOH was the precursor of the ND-mTHPP, its cytotoxicity was also evaluated. The cytotoxicity of ND samples was evaluated by using cells cultured in the absence of samples and irradiation as reference and the values were discussed according to the established value of 70% of cell viability as a minimum value to consider a material as noncytotoxic (ISO 10-993-5) [48].

**Figure 7:**
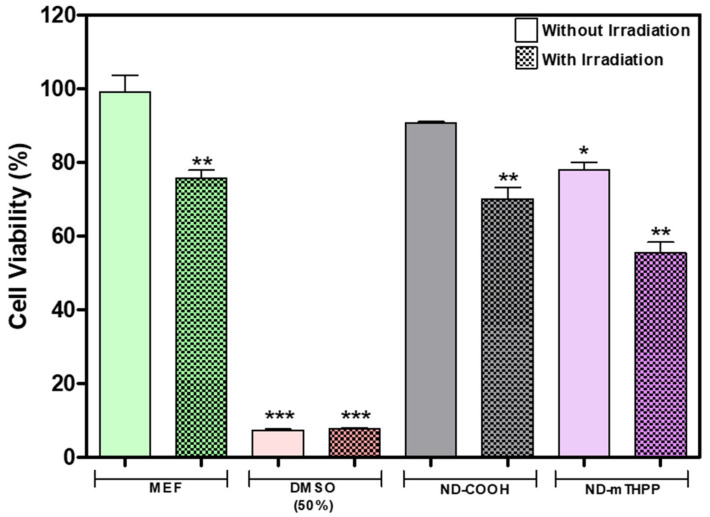
Cytotoxicity ND-COOH (100 μg/mL) and ND-mTHPP (100 μg/mL) in MEF culture after 24 h, with and without irradiation. Cell viability of ND-COOH and ND-mTHPP measured using MTT assay. Experiments were repeated in triplicate and significant differences between MEF control (without irradiation) and test samples are marked with * (*p* < 0.05), ** (*p* < 0.01), and *** (*p* < 0.001).

The viability of MEF cells (Figure 7) incubated with ND-COOH at 100 μg/mL was high enough (90.7 ± 0.3%) to consider this sample as noncytotoxic. In comparison with this result, the viability of MEF cells incubated with ND-mTHPP was lower (78 ± 2%) but still in the range that allows the classification of this sample as noncytotoxic. Since in the cells incubated with ND-COOH the effect of irradiation was not significant and the cell viability was close to the value found for cells incubated in the absence of irradiation, it was possible to conclude that the low viability of cells incubated with ND-mTHPP and irradiated (56 ± 5%) was a result of mTHPP photoactivity. 

Although the results indicated that ND-mTHPP is cytotoxic under irradiation, the absence of cytotoxicity of ND-mTHPP in the absence of irradiation was a positive feature of this nanoconjugate and was considered an encouraging result to its application as antimicrobial agent. Even if when in the biological medium the ND-mTHPP may come in contact with mammalian cells, it would not be expected to cause severe toxic effects as long as the irradiation would be punctually directed to the target tissue wherein abnormal bacterial growth would have been identified. 

## 4. Discussion

A large effort in the field of nanoparticles has been dedicated to the development of nanoplatforms with enhanced efficiency and broader spectrum of antimicrobial activity. In this context, the coupling of antimicrobial agents with ND has been a clever strategy to obtain highly efficient nanoconjugate since ND are already known to have antimicrobial activity depending on their surface chemistry and size. Herein, the development of a ND-based nanoconjugate aimed its application in aPDT. The porphyrin mTHPP was conjugated with carboxylated ND and the resultant nanoconjugate ND-mTHPP was evaluated regarding its antibacterial activity and cytotoxicity against MEF cells.

Although noncovalent bonds have been widely used to couple bioactive molecules with ND [49,50,51,52], this method shows the disadvantage of low reproducibility and liability of the ND-molecule bond, which can be a result of nonhomogeneous ND surface chemistry combined with the low colloidal stability of ND. The surface functionalization of ND mainly with reactive carboxyl and hydroxyl groups brought the possibility of conjugation with a diversity of chemical groups or molecules (dyes, monomers, polymers, proteins, photosensitizers) [40,53,54], by covalent bond. Amongst the further chemical reaction that carboxylated ND can go through, the esterification outstands for providing increased colloidal stability of ND in aqueous suspension [40,55,56,57]. In this work, the nanoconjugate ND-mTHPP was obtained by an ester bond between carboxylated ND and mTHPP. The ND-mTHPP proved to be stable in aqueous suspension, showing small particle size and narrow size distribution which are suitable features for bioapplications.

ND-mTHPP showed a prominent antibacterial activity in the initial bacterial growth phase (2 h) when the inhibition of *E. coli* growth was 42 ± 6% in the absence of irradiation. The antibacterial effect was 14% higher under irradiation (660 nm; 51 J/cm^2^) and the ND-mTHPP provided 56 ± 6% of inhibition of bacterial growth. Notably, the inhibitory activity of the antibiotic ampicillin was much lower (26 ± 6%) at the same conditions. Ampicillin was shown to be more efficient only after 7 h of incubation, when its inhibitory activity was of 65 ± 3% whereas ND-mTHPP showed 33 ± 6% of inhibition. This difference between the incubation time for optimal inhibitory activity could be afforded by the different mechanism of action of each agent. Ampicillin is well-characterized to act by inhibition of bacterial cell wall synthesis and therefore its effect would increase with bacterial growth. ND-mTHPP would act by inducing bacterial cell wall damage as evidenced by SEM images and by ROS generation under irradiation due to the photoactivity of mTHPP. In this case, the lower inhibitory activity after 2 h of incubation could be a consequence of the deactivation of ROS and the recovering ability of the remaining bacteria. Herein, the irradiation was performed only once and right after the incubation. We believe that the efficiency of the treatment with ND-mTHPP could be improved by performing more than one irradiation, periodically after each 2 h, in order to keep active ROS at suitable concentration levels and to avoid bacterial recovering. The optimal activity of the ND-mTHPP during the initial phase of bacterial growth can be an advantage of this system over the conventional antimicrobial agents, since the bacterial growth could be controlled before the development of an infectious disease. Probably, the antibacterial activity of the ND-mTHPP would be compromised at higher bacterial density since bacteria can change the composition of the medium during its growth by excretion of metabolites and ions. The increased concentration of salts and ions could reduce porphyrin photoactivity since some ions, especially metallic ions, are known to form complexes with porphyrins [58,59,60]. For future considerations, it should be also mentioned that at high density of bacteria, the medium would become more turbid, increasing the effect of light scattering that would hamper the irradiation of ND-mTHPP later than right after the incubation, as performed in this work.

Despite the distinct methods of analysis of bacterial growth and of ND size characterization (TEM or DLS), it was possible to state that in comparison with the results reported before regarding antimicrobial activity of ND, the nanoconjugate ND-mTHPP showed better performance. Lyer et al. (2018) [45] reported 50% of growth inhibition of uropathogenic *E. coli* (UPEC) by the bacterial exposition to 6 nm (measured by TEM) detonation ND at 200 μg/mL for 2 h. Although ND-mTHPP provided a similar inhibitory effect (56 ± 4%), it has to be highlighted that this result was obtained by using the nanoparticles at half of the concentration (100 μg/mL). Wheling et al. (2018) [19] observed an inhibitory effect after shorter incubation time (15 min) by using 60 nm detonation ND (measured by DLS) but at a higher concentration (500 μg/mL). The ND provided 90% of inhibition of *E. coli* K12 (DSMZ No. 1077) which was calculated by measurements of bacterial ATP levels. By using ND and methodology similar to those used in our work, Chatterjee et al. (2016) [18] reported on the antibacterial activity of 5 nm detonation ND (measured by TEM). The growth of *E. coli* (HB101) was monitored by OD_600_ measurements for 13 h. They observed a delay in bacterial growth in the first 3 h by adding ND at 100 μg/mL. The authors also reported lower antibacterial effect of ND agglomerates of 100 nm which were identified on bacterial cell wall by SEM. Similar agglomerates were also observed to be formed by ND-mTHPP on bacterial cell wall but differently from what they observed, these agglomerates of ND-mTHPP were still able to induce bacterial death by membrane disruption as suggested by SEM images.

The antibacterial activity of ND-mTHPP was in line with the results reported before regarding ND conjugated with photoactive molecules. Openda et al. (2020) [61] performed noncovalent conjugation of metallo-phthalocyanine complexes with detonation ND through π-π stacking interaction and evaluated the aPDT activity against *S. aureus* CFU counting. Although they reported on inhibitory activity of bacterial growth at 9.72 on the log10 scale by using the conjugated at a very low concentration (10 μg/mL) the irradiation dose was much higher (943 J/cm^2^) than the irradiation dose applied in the present study (51 J/cm^2^). Moreover, since the conjugation reported by Openda et al. (2020) [61] was by adsorption and not by covalent bond, phthalocyanine could be released in the medium. Therefore, it would be difficult to distinguish if the inhibitory activity was a result of the conjugate or also of the free phthalocyanine. 

Another important feature presented by the nanoconjugate ND-mTHPP was the low cytotoxicity against MEF cells in the absence of irradiation. The viability of MEF cells cultured in the presence of ND-mTHPP at 100 μg/mL was 78 ± 2%. Accordingly, Gismondi et al. (2015) [62] also reported cell viability values greater than 80% after incubation of B16-F10 and HeLa cells with detonation ND of 4–5 nm at 200 μg/mL. The low cytotoxicity of ND was also reported in our previous work (Hurtado 2020) [12]. ND of 22–77 nm diameter were obtained from micrometric diamond HFCVD by ultrasonic cavitation. When MEF cells were cultured in the presence of these ND at 116 μg/mL they showed viability of 70%. Higher cytotoxicity was observed by using lower ND concentration which allowed us to infer that the cytotoxicity was a result of the ND interaction with cell membrane and therefore it would depend on size, colloidal state and surface chemistry and composition. As highlighted by the DLS measurements, ND-mTHPP aggregates in DMEM medium and, therefore, colloidal stability of nanoparticles suspension is compromised as concentration increases. Therefore, at low concentration ND would interact with cell membrane smaller nanoparticles which, according to several researchers, are able to perforate the cell membrane, being inserted or inducing the formation of multiple pores. However, larger nanoparticles (≈100 nm), such as the ND-mTHPP agglomerates formed at high concentration, have been shown to be covered by the lipid bilayer, being incorporated into the membrane, without inducing pore formation. Consequently, lower cytotoxic effects are observed with nanoparticles at this size range. 

Similar to what was observed in the antibacterial assay, the presence of irradiation (660 nm; 51 J/cm^2^) decreased the viability of cells incubated with ND-mTHPP. The photoactivity of this nanoconjugate was already expected to be observed not only against bacteria but also against the mammalian cells, such as MEF, since although the interaction with cell membrane and bacterial cell wall may differ, the mechanism of action of irradiated mTHPP by ROS generation can damage both types of cells. Indeed, the application of ND has been increasingly explored in PDT and some works have shown that enhanced photoactivity can be afforded by coupling photosentizers with ND. The photoactivity of ND-based nanoconjugates has already been addressed by Matshitse et al. (2020) [38]. The cytotoxicity of ND conjugated with (2,9,16,23-tetrakis[4-(N-methylpyridyloxy)]-phthalocyanine (ZnTPPcQ) at 50 μg/mL was evaluated against MCF-7 breast cancer cells. The authors also evaluated the cytotoxicity of the raw material (ND). Surprisingly, although both conjugated ND and bare ND had showed no cytotoxicity in the absence of irradiation, when the cells were irradiated (13.5 J/cm^2^), not only the conjugate but also the bare ND showed intense photoactivity. The viability of cells incubated with ND was 30% and the cells incubated with the conjugate showed 29% of viability under irradiation. Another interesting application has been pointed out by the studies on the interaction of ND with light. Some works have shown a photoprotective effect of ND against UV light. Wu et al. (2015) [63] investigated the protection of MEF cells under incubation with 2 mg/cm^2^ of ND of 5 and 100 nm. An effective protection against an irradiation dose of 68 mJ/cm^2^ of UVB irradiation was observed in the presence of 5 nm ND (73%) and 100 nm ND (82%). 

Considering the prominent antibacterial activity against *E. coli* and the low cytotoxicity against MEF cells, it is expected that the nanoconjugate ND-mTHPP can find application in aPDT as potential antimicrobial agents. The authors believe that this work will encourage further investigation on the application of this system for controlling the growth of other strains of microorganisms.

## 5. Conclusions

The results presented in this work give us a promising focus for ongoing research. It was shown that conjugation of photosensitizer with ND can be explored as a strategy to improve ND colloidal stability and to combine photodynamic activity with ND antimicrobial activity. The nanoconjugate ND-mTHPP is expected to promote enhanced antimicrobial activity not only against *E. coli* but also against other strains of microorganisms. From the best of our knowledge, this is the first work that reports on the covalent binding of a porphyrin to ND and proves that it is an effective platform for inhibition of *E. coli* growth without inducing significant cytotoxicity against mammalian cells. Therefore, the ND-mTHPP developed in this work shows potential application in aPDT.

## Figures and Tables

**Table 1:** Hydrodynamic diameter of ND-mTHPP in aqueous suspension, DMEM and MHB at 100 μg/mL. * Value statistically different from the others (*p* < 0.05).

Solvent	D (nm)
Water	58 ± 1
Mueller Hinton Medium	58 ± 2
DMEM Medium	82 ± 3 *

**Table 2:** Inhibition of *Escherichia coli* growth by ND-mTHPP, with and without irradiation, calculated from 0 to 24 h.

	Inhibition *E. coli* Growth (%)	
Incubation Time (h)	Without Irradiation	With Irradiation
0	24 ± 14	24 ± 7
0.5	33 ± 7	29 ± 3
1	36 ± 6	43 ± 3
1.5	42 ± 7	50 ± 3
**2**	**42 ± 6**	**56 ± 4**
2.5	39 ± 8	46 ±4
3	40 ± 8	47 ± 3
5	32 ±8	50 ± 3
7	33 ± 6	37 ± 4
24	27 ± 6	31 ± 5

## Data Availability

Raw data were generated at ICT-UNIFESP. Derived data supporting the findings of this study are available from the corresponding author D.T. on request.
